# Circular Economy of Coal Fly Ash and Silica Geothermal for Green Geopolymer: Characteristic and Kinetic Study

**DOI:** 10.3390/gels8040233

**Published:** 2022-04-11

**Authors:** Himawan Tri Bayu Murti Petrus, Muhammad Olvianas, Muhammad Faiz Shafiyurrahman, I Gusti Agung Arvin Nanda Pratama, Siti Nurul Aisyiyah Jenie, Widi Astuti, Muhammad Istiawan Nurpratama, Januarti Jaya Ekaputri, Ferian Anggara

**Affiliations:** 1 Department of Chemical Engineering (Sustainable Mineral Processing Research Group), Faculty of Engineering, Universitas Gadjah Mada, Jalan Grafika 2, Yogyakarta 55281, Indonesia; bayupetrus@ugm.ac.id (H.T.B.M.P.); muhammad.olvianas@mail.ugm.ac.id (M.O.); muhammad.faiz.s@mail.ugm.ac.id (M.F.S.); arvin.nanda@mail.ugm.ac.id (I.G.A.A.N.P.); 2 Unconventional Geo-Resources Research Group, Faculty of Engineering, Universitas Gadjah Mada, Jalan Grafika 2, Yogyakarta 55281, Indonesia; 3 Konsorsium Riset Geopolimer Indonesia, NASDEC, Kampus ITS, Sukolilo, Surabaya 60111, Indonesia; januarti@ce.its.ac.id; 4 Research Centre for Chemistry, National Research and Innovation Agency (BRIN), Kawasan Puspiptek Building 452, Serpong, Tangerang Selatan 15314, Indonesia; siti043@brin.go.id; 5 Research Center for Mining Technology, National Research and Innovation Agency (BRIN), Jl. Ir. Sutami Km. 15, Tanjung Bintang, Lampung Selatan, Kota 35361, Indonesia; widi005@brin.go.id; 6 PPT Geo Dipa Energi (Persero), Jalan Warung Jati Barat No.75, Kecamatan Pancoran, Jakarta Selatan 12510, Indonesia; istiawan@geodipa.co.id; 7 Department of Civil Engineering, Faculty of Civil, Planning and Geo Engineering, Institut Teknologi Sepuluh Nopember, Kampus ITS, Sukolilo, Surabaya 60111, Indonesia; 8 Department of Geological Engineering, Faculty of Engineering, Universitas Gadjah Mada, Jalan Grafika 2, Yogyakarta 55281, Indonesia

**Keywords:** fly ash, geothermal silica, dry activator, geopolymer, kinetics

## Abstract

The study of geopolymers has become an interesting concern for many scientists, especially in the infrastructure sector, due to having inherently environmentally friendly properties and fewer energy requirements in production processes. Geopolymer attracts many scientists to develop practical synthesis methods, useful in industrial-scale applications as supplementary material for concrete. This study investigates the geopolymerization of fly ash and geothermal silica-based dry activator. The dry activator was synthesized between NaOH and silica geothermal sludge through the calcination process. Then, the geopolymer mortar was produced by mixing the fly ash and dry activator with a 4:1 (wt./wt.) ratio. After mixing homogeneously and forming a paste, the casted paste moved on to the drying process, with temperature variations of 30, 60, and 90 °C and curing times of 1, 3, 5, 7, 14, 21, 28 days. The compressive strength test was carried out at each curing time to determine the geopolymer’s strength evolution and simulate the reaction’s kinetics. In addition, ATR-FTIR spectroscopy was also used to observe aluminosilicate bonds’ formation. The higher the temperature, the higher the compressive strength value, reaching 22.7 MPa at 90 °C. A Third-order model was found to have the highest R^2^ value of 0.92, with the collision frequency and activation energy values of 1.1171 day^−1^ and 3.8336 kJ/mol, respectively. The utilization of coal fly ash and silica geothermal sludge as a dry activator is, indeed, an approach to realize the circular economy in electrical power generations.

## 1. Introduction

Electricity is one of the most vital basic needs for daily activities; therefore, many power plants are built to fulfil this growing demand. The coal-burning power plant is still widely used in developing countries, such as Indonesia, because the plant operation is well established, and coal supplies are both abundant and economical [1]. However, 15–35% bottom ash and 65–85% fly ash would be produced from the total coal combustion byproduct (CCB) residue [2]. Fly ash contains silica, alumina, and several heavy metals, such as iron, magnesium, titanium, manganese, nickel, chromium, mercury, arsenic, and copper, with 0.5–200 microns [3,4,5]. Ash waste from coal combustion is generally disposed of in landfills; thus, the ecosystems around disposal areas and industries, such as forests, rivers, and swamps, have experienced the adverse effect of this pollution [6]. The utilization of fly ash as a construction material has been widely applied in the construction industry due to economic profitability, as cement can be partially replaced by fly ash and have beneficial effect on concrete production [7]. Fly ash can be used as an additive to Ordinary Portland cement (OPC) [8]. Silica (Si) and alumina (Al) content in fly ash increase its potential use as a geopolymer material. More efforts have been reported to exploit fly ash applications, such as a Fenton-like catalyst [9], organic adsorbent [10], alternative sources of rare earth elements [11,12,13], and cenospheres [14,15,16].

Geopolymers have been widely studied and developed by mixing Al-Si rich-fly ash with high alkaline solutions as an activator [17,18,19]. The SiO_2_/Al_2_O_3_ ratio in the ideal pozzolan for composing geopolymer concrete is around 3.0–3.8 [20,21,22]. There are many shortcomings in handling, storing, and applying geopolymers produced using a wet activator, as shown in Table 1. Many researchers have started to develop dry activators to replace wet processes [23]. Solid activators provide alkaline cations that can dissolve the Si and Al monomers present in fly ash particles [24]. Some chemicals can be used as solid activators, such as sodium hydroxide, sodium silicate, sodium aluminate, sodium carbonate, sodium sulfate, and potassium hydroxide [23,25]. Sodium silicate was used as the solid alkaline activator in this study. Practically, sodium silicate can be synthesized from NaOH and silica-containing materials, such as geothermal sludge, through a calcination process at 400 °C, the optimum calcination temperature to produce green geopolymer mortar [23]. Hence, the energy requirement is much less than the limestone and clay calcination during OPC production [26]. Further, environmental pollution due to limestone’s calcination process will produce carbon dioxide emissions of 0.8–1.35 tonnes of CO_2_ per tonne of clinker produced [27].

Geothermal sludge formed in the upstream pipe carrying water from the geothermal reservoir to the surface can reduce power plant performance. Thus, the silica scaling needs to be discharged from the upstream pipe during the maintenance period. The waste produced at the geothermal power plant should be managed appropriately to prevent the negative impact of environmental pollution. Amorphous silicon dioxide (SiO_2_) is the main component in geothermal scaling waste, comprising 90–98% of the total mass, which is compatible as a starting material for alkaline activator production [32]. The use of silica sludge can be an innovative solution to increase the eco-friendliness of geothermal power plants. Furthermore, it can also increase revenue and reduce geopolymer production costs [33]. Developing geopolymer as green concrete is enticing, mainly because the CO_2_ emissions produced in overall production activities are 10% lower than OPC, reaching 354 kg CO_2_- eq/m^3^ [28].

Geopolymers synthesized from class F fly ash, and geothermal silica-based dry activators, have been previously studied by [23]. However, to this date, there is no report on the kinetics of geopolymer activated by a calcination product of geothermal sludge and sodium hydroxide powder. The kinetic models used in this study are written in Table 2. Geopolymerization is well known as a complex and multicomponent-dependent reaction. The solid-state kinetic models can be used to understand the reaction mechanism of a complex reaction system. The Avrami kinetic model is mainly used to interpret the nucleation process. In geopolymer materials, the silicon and aluminium monomers are cross-linked, leading to the formation of a 3D geopolymer structure. The growth of the geopolymer gel structure could be identical to the nucleation process, which can be explained by the Avrami kinetic model [33]. The geometric contraction model assumes that the growth of the oligomer would rapidly occur on the particle’s surface [34]. As the raw materials of geopolymer are mostly fine spherical particles, there is a possibility that geopolymer growth would follow the shape of raw material particles. The diffusion and third-order reaction models are closely related to the reactant mass dynamics, such as reactant transport, concentration, and consumption rate [34].

This report studied the impact of dry activators on geopolymer kinetics. The geopolymer mortars made of class C fly ash and geothermal silica-based dry activators were prepared and mechanically tested. The kinetics models were studied using the compressive strength correlation approach, assuming the material strength is strongly correlated to the quantity of the aluminosilicate bond in the geopolymer. The development of the aluminosilicate bond was also observed by Attenuated Total Reflectance-Fourier Transform Infra-Red (ATR-FTIR) spectroscopy. Understanding the characteristics and kinetics of the geopolymer made of coal fly ash and silica geothermal sludge as dry activators will surely benefit the production scaling up and provide economic added value to these two solid side-products from their electric generation plants to create a circular economy.

## 2. Materials and Methods

### 2.1. Materials

Class C fly ash was obtained from PT. Paiton Energy, Power Plant, Indonesia. Geothermal sludge was obtained from PT Geo Dipa Energi, a geothermal power plant located in Dieng, Central Java, Indonesia. Sodium hydroxide flakes (95% technical grade) were purchased from Merck.

### 2.2. Dry Activator Synthesis

**Figure 1:**
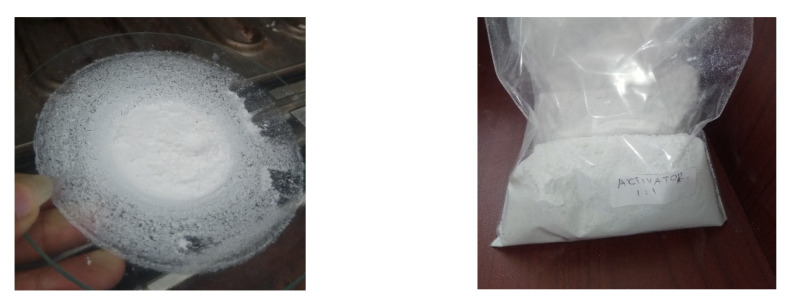
Dry activator synthesized by mixing NaOH and geothermal silica with a ratio of 1:1 (wt./wt.) and then calcining at 400 °C.

Geothermal sludge was dried at 150 °C for 7 h. The dried sludge was mashed then sieved with a sieve machine to obtain a size of −200 mesh. Then, sodium hydroxide (NaOH) and geothermal sludge with a mass ratio of 1:1 were mixed and pulverized with a mortar and pestle. The mixture was then put into a furnace, and the temperature was set at 400 °C for 2 h at a heating rate of 10 °C/min. The dry activator was refined and sieved to obtain the size of −200 mesh particles (Figure 1).

### 2.3. Production of Geopolymer Mortar

**Figure 2:**
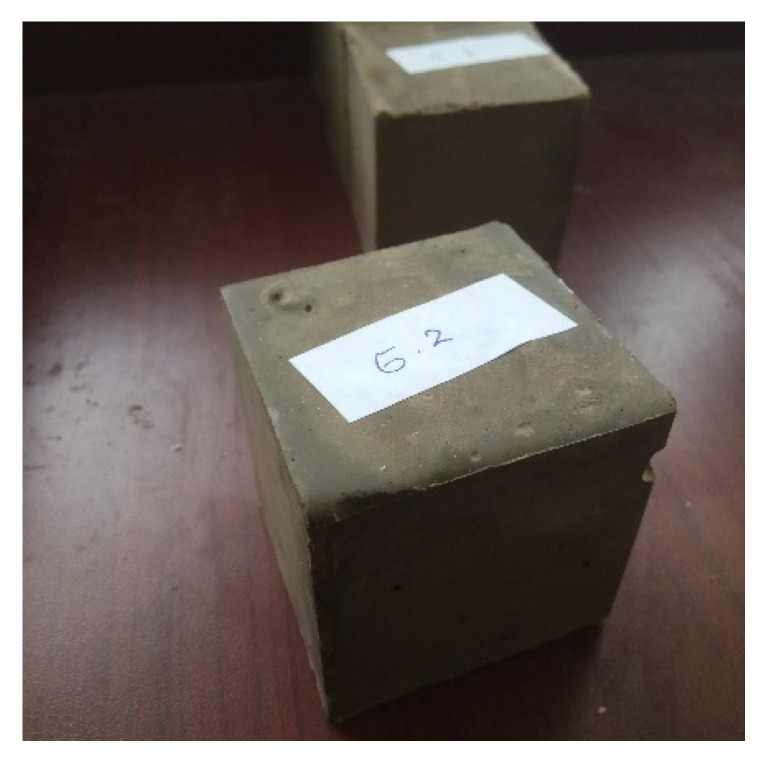
Mortar geopolymer made of coal fly ash and dry activator from silica geothermal sludge.

Geopolymer mortar production was started by mixing 580 g of fly ash and 145 g of dry activator (Figure 2). After both ingredients were mixed, water was added to 20% of the total solid mass. Water was poured gradually while being stirred until it formed a paste. The geopolymer paste was moulded on a 5 × 5 × 5 cm^3^ cube mould. The casted geopolymer paste was allowed to stand idle for two days; then, it was removed from the mould. Drying was carried out at 30 (room temperature), 60, and 90 °C.

### 2.4. Instrumental Analysis

The geopolymer mortar samples’ compressive strength was tested using the Avery-Denison Universal Testing Machine for each curing time-variant (1, 3, 5, 7, 14, 21, and 28 days). The same test steps were performed for geopolymer concrete at curing temperatures of 60 and 90 °C. Oxide component analysis of geothermal sludge and fly ash was carried out using Energy Dispersive X-ray Spectroscopy (EDX-8000 Shimadzu) to determine its constituents, with operating conditions such as atmospheric air, collimator 10 mm with the analyte for fly ash in the form of Al-U and C-Sc and geothermal sludge in the form of Na-U. The phase analysis of the dry activator was carried out with X-ray Diffraction Spectroscopy (XRD) PANalytical X’pert 3 powder using Cu-Kα irradiation. Attenuated Total Reflectance-Fourier Transform Infra-Red (ATR-FTIR) Spectroscopy Nicolet iS5 was used to observe the formation of aluminosilicate bonds in geopolymer samples.

## 3. Results and Discussion

### 3.1. Raw Materials

Raw material characterization is crucial to determine the potential use of fly ash and geothermal sludge as a geopolymer. Fly ash was tested for chemical analysis with EDX to determine its chemical composition, especially Si and Al, which play an essential role in the geopolymerization reaction. Based on Table 3, fly ash used in this study can be categorized as type C fly ash due to having a Si/Al ratio of 3, which is in a good range (Si/Al: 3.0–3.8) to produce good material strength [15,16,17].

Geothermal sludge was also analyzed using EDX to determine its oxide composition. Based on the geothermal silica analysis results after drying and sieving, the silica content was 99.52%, as shown in Table 4. The high purity of silica means that the geothermal sludge does not need further purification steps.

### 3.2. Dry Activator

**Figure 3:**
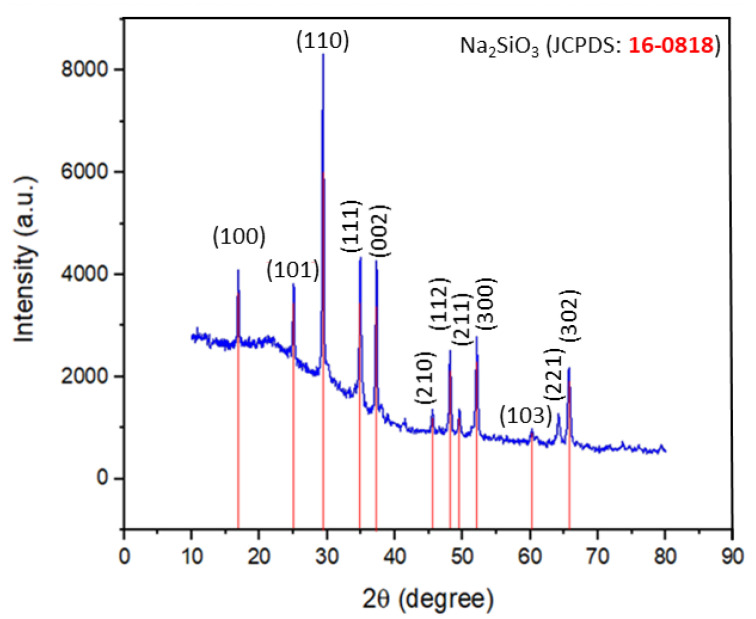
XRD pattern of dry activator synthesized by mixing NaOH and geothermal silica with a ratio of 1:1 (wt./wt.) and then calcining at 400 °C.

During the calcination process, the sodium hydroxide would melt at 318 °C and fuse to the geothermal silica to form sodium metasilicate. The crystallinity of the products can be controlled by adjusting the calcination temperature and Na_2_O/SiO_2_ ratio [18,30]. Based on Figure 3, the XRD pattern of the dry activator has a similar pattern with hexagonal sodium metasilicate (Na_2_SiO_3_) (JCPDS: 16-0818). The major peaks at 16.8°, 24.9°, 29.3°, 34.8°, 37.4°, 48.1°, 52.0°, and 65.7° were indexed to the (100), (101), (110), (111), (002), (112), (300), and (302) planes. Although the XRD pattern is highly matched with crystalline planes, the XRD pattern also shows a hump in a range of 15° to 35° diffraction angle (2θ), which indicates the existence of an amorphous phase in the mixture. Based on the XRD analysis of the activator in Table 5, the fraction of the amorphous phase was found to be 78.86%, while the Na_2_SiO_3_ crystalline phase was about 21.14%. This is supported by [23], in their study on green geopolymer, which shows the optimal activator ratio of NaOH/Geothermal Silica is 1:1 at 400 °C, with a Na_2_O/SiO_2_ ratio of 0.692. A similar result has also been reported on sodium silicate synthesis from glass cullet and NaOH using a reaction temperature of 450 °C, with estimated amorphous content of 75% [35]. Dry geopolymer production has a slower reaction due to additional steps of alkaline cation formation, interrupting the digestion step of Si–Al minerals originating from raw materials. This alkaline cation will release silica and alumina monomers [18].

Geopolymerization in a dry activation system generally involves several simultaneous steps, i.e., alkaline cations formation, mineral dissolution, oligomerization, polycondensation, and stabilization [36]. In the alkaline cation formation stage, the process is strongly influenced by the presence of water. This geopolymer system can be divided into non-evaporable and evaporable water. According to [37], non-evaporable water is chemically bound, which results from the surface hydroxylation of silica, whose amount will decrease with increasing NaOH concentration and evaporate above 100 °C. In contrast, evaporable water is the water trapped between geopolymer structures and evaporates under a temperature of 100 °C, continuing the dissolution stage; that is, the dissolving of solid aluminosilicate into silica monomers and alumina in alkaline solid cations. The process is continued by forming an oligomer of Si and Si–Al in the liquid phase, followed by the polycondensation of species or oligomer units in the water phase to form inorganic polymer materials. The last stage bonds the unreacted solid materials in the geopolymer structure, followed by structural stabilization [38].

### 3.3. Compressive Strength

The time-dependent-compressive strength of geopolymers can be used to simulate the kinetic processes, as the quantity of aluminosilicate bond strongly correlates with the material strength. The synthesis temperature highly influences the rate of polymerization of aluminosilicate formation. In [33], the authors show that a higher curing temperature could accelerate the kinetics of geopolymer produced by wet activation methods. Another report by [39] presents the geopolymer kinetics models using wet activators. The study indicates that the compressive strength test correlates with the number of aluminosilicate bonds, as shown by the FTIR CORR value. 

The curing temperature variations used were 30, 60, and 90 °C, with the period of the geopolymer concrete curing process at seven points in time; specifically, on 1, 3, 5, 7, 14, 21, and 28 days. There was an insignificant increase in compressive strength on days 14 and 28; thus, the test with the longest drying time was 28 days, with the expectation that the geo-polymerization reaction was considered optimum. Following are the results of the compressive strength analysis of geopolymer concrete.

**Figure 4:**
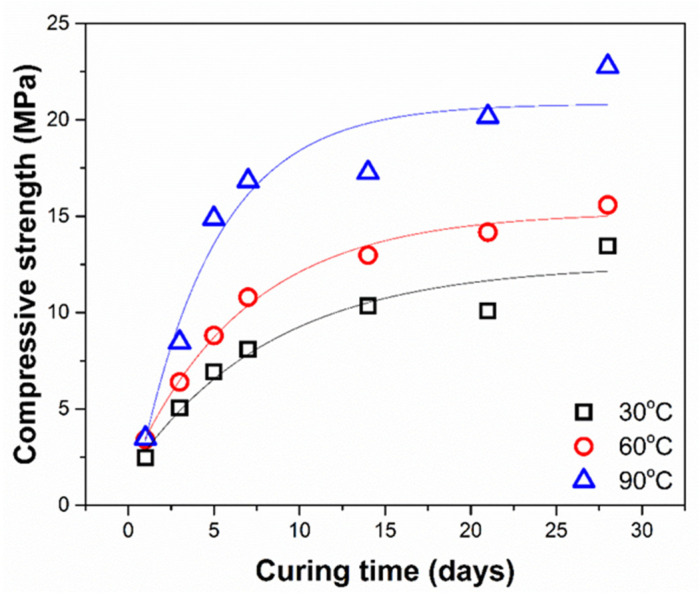
Compressive strength of geopolymer mortars cured at different temperatures.

Based on Figure 4, it can be seen that, at various curing temperatures, the concrete has the same overall trend, where the compressive strength value increases with a longer curing time. However, the compressive strength will rapidly reach the optimum value on the 14th day and slowly increase until the maximum values at the curing time of the 28th day. Even though all measured compressive strength values show increasing trends, there is a prominent difference due to curing temperatures. A higher curing temperature gives faster reaction kinetics, indicated by a sudden increase in compressive strength within a short curing time. As shown in Figure 4, a curing temperature of 90 °C gives a steep profile curve, both in compressive strength and degree of reaction.

On the other hand, a lower curing temperature (30 °C) has a curvier profile, indicating a lower kinetics rate. The water release coincides as the material strength increases due to the aluminosilicate bond formation. Increasing the drying temperature can accelerate the rate of evaporation and increase the value of compressive strength.

The curing temperature greatly affects the formation of Si–O–Si or Si–O–Al bonds from Si–OH bonds, providing stability and increasing strength. However, the higher temperature will impact the speed of the evaporation process, which can lead to the formation of larger pores and result in cracking in the concrete. Due to water evaporation in a concrete mixture, cracks are called plastic cracking [40]. Minimizing the risk of increasing the cracks’ size can be done by reducing the drying heat rate; thus, the speed of H_2_O evaporation can be controlled from the pores of the sample to the sample surface.

### 3.4. ATR-FTIR Analysis and Effect of Curing Temperature on Geopolymer Kinetics

**Figure 5:**
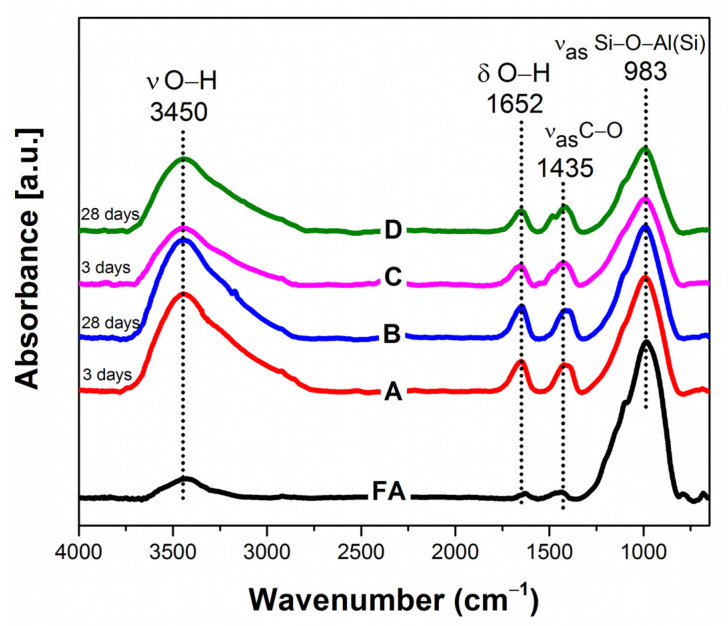
ATR-FTIR spectra of class C fly ash and selected geopolymer samples with curing temperature of (**A**,**B**) 30; and (**C**,**D**) 90 °C.

Figure 5 shows FTIR spectra of class C fly ash and selected geopolymer samples cured for 3 and 28 days, under a temperature-controlled environment (30–90 °C). As can be seen, the fly ash has a different band shape, especially at the wavenumber of 3450, 1652, and 1435 cm^−1^. The fly ash spectrum has a lower intensity compared to the geopolymer samples. The absorption band in 3450 and 1652 cm^−1^ are detected due to the stretching vibration of the H–OH bonds in the chemically bonded water [41]. The intensity changes on the absorption band of 3450 and 1652 cm^−1^ indicate that the hydration process occurred in the fly ash and dry activator mixtures. The strong absorption band at 1435 cm^−1^ is observed due to sodium carbonate (Na_2_CO_3_) [42], suggesting that the sodium ions from dry activators were partially reacted with atmospheric CO_2_ gas during the curing process.

The prominent bands at 983 cm^−1^ are observed in fly ash and geopolymer samples. However, geopolymer samples have lower intensities and different shapes than the fly ash band. This absorption band corresponds to the asymmetric stretching vibration of the Si–O–T (T: tetrahedral Si or Al) bond [43]. The sharp peak of fly ash at 983 cm^−1^ is transformed into a broad peak, found in geopolymer spectra. This finding implies that Si–Al minerals in fly ash are partially dissolved to form Si–Al monomers. The crystalline phase in fly ash gives a sharp rather than a broad peak, as shown by amorphous geopolymer [44]. The broadening phenomenon on Si–O–T asymmetric bands indicates multiple overlapping components [45].

**Figure 6:**
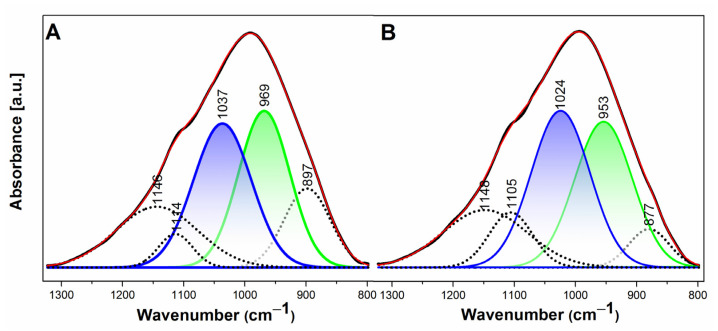
Deconvolution of ATR-FTIR spectra of selected geopolymer samples with curing time of 28 days and curing temperature of (**A**) 30; and (**B**) 90 °C.

Deconvolution analysis within the vibration range of 798–1323 cm^−1^ of ATR-FTIR spectra is provided to unveil and comprehend the influence of curing temperature on geopolymer kinetics. The deconvolutions of spectra were carried out using the OriginPro software with Gaussian peak shapes and following the Levenberg–Marquardt (L-M) algorithm. The procedure of deconvolution spectra is similar to a previous report by [45]. Figure 6 illustrates that the deconvoluted spectra consist of five bands corresponding to the specific components. The bands at 877–897 cm^−1^ are associated with the OH bending in Si–OH groups, which can be assumed from unreacted activating solutions [45]. The absorption bands at 953–969 cm^−1^ are due to non-bridging oxygen (NBO) stretching in the Si–O–Na structure, associated with immature aluminosilicate bonds [45]. The asymmetric stretching Si–O–T (T: tetrahedral Si or Al) is located at a wavenumber of 1024–1037 cm^−1^, which links to the geopolymer networks [46,47], while the absorption bands at 1105–1148 cm^−1^ are linked with asymmetric stretching Si–O–T (T: tetrahedral Si or Al) from unreacted fly ash [45]. 

By assuming that the relative area under curves is proportional to the concentration, interesting information can be generated from this deconvolution study. As shown in Figure 6, the normalized area of the NBO stretching absorption band for geopolymer cured at 30 °C has a larger area than the geopolymer cured at 90 °C. The NBO stretching absorption band area decreases as curing temperature increases, while the Si–O–T geopolymeric band increases. This suggests that curing temperature could accelerate the reaction kinetics.

### 3.5. Geopolymerization Kinetics

**Figure 7:**
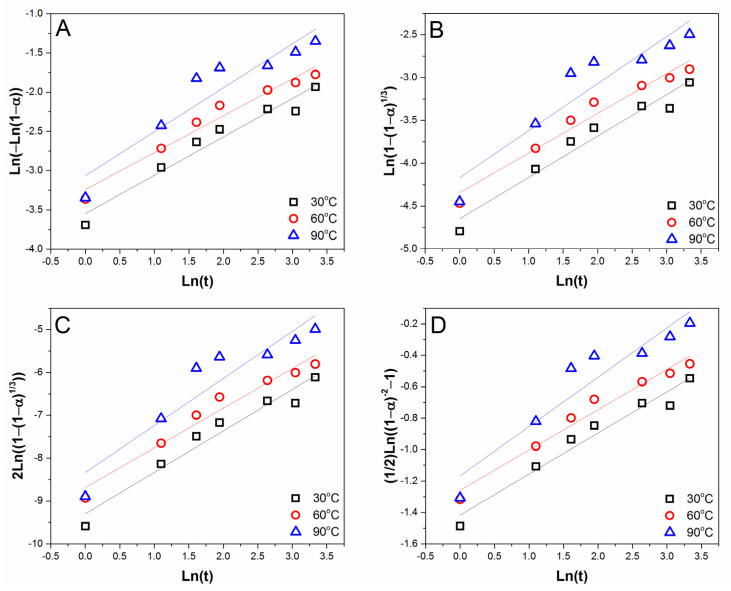
Graph of models fitting with (**A**) Avrami; (**B**) Geometric Contraction; (**C**) Diffusion; (**D**) Third order equation and determination of their constant reaction values.

The compressive strength test results were converted into the degree of reaction (α). The degree of reaction is a dimensionless quantity that can be determined as the ratio between compressive strength at a specific curing time, with “assumed” maximum compressive strength [33]. In our case, the mortar’s strength is higher than 1 MPa, and below 100 MPa. Hence, the strength was divided by 100. The compressive strength values that have been normalized into the degree of reaction were then substituted into several kinetic models; particularly, Avrami, geometric contraction, diffusion, and third-order kinetic model, as shown in Figure 7. The most suitable kinetics model for the geopolymerization reaction using the dry method can be determined by comparing the R-Square value of each model.

**Figure 8:**
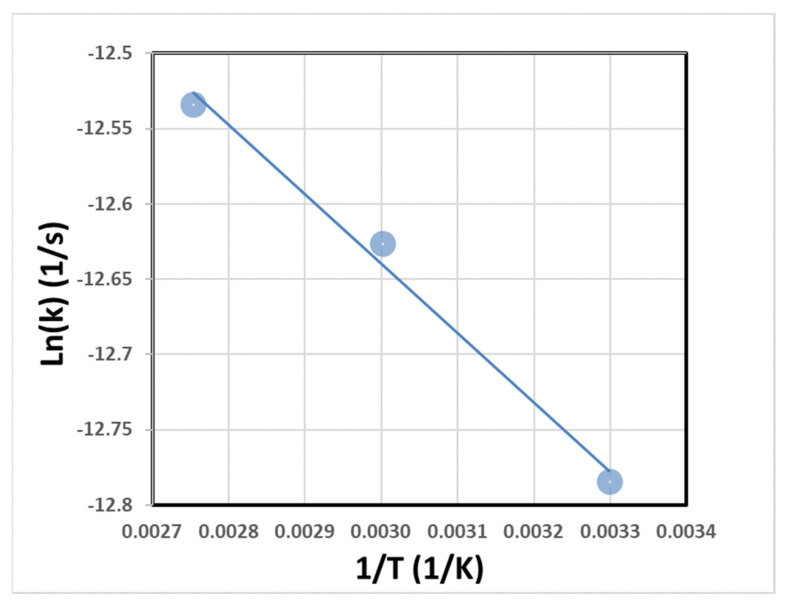
The temperature dependence of reaction kinetic constant from the third-order kinetic model.

Based on Table 6, it can be concluded that the kinetics model that is most suitable for the geopolymerization reaction is the third-order model, with the mean R-square value of 0.9448, at a range of 30 to 90 °C. The result implies that geopolymerization may be influenced by the reactant concentration and consumption rate. The diffusion kinetics model also has a high possibility of describing the geopolymerization process, as the alkaline solutions will diffuse into the particle pores [38]. Further, in other studies, it is stated that the diffusion kinetics model is the most widely used model for pozzolanic reactions [48]. The other important information gathered from Figure 7 is the reaction constants. Following the Arrhenius equation, these values can determine the apparent activation energy (E) and the collision frequency (A). The logarithm data of reaction constant from three isothermal temperatures against the inverse of absolute temperatures is shown in Figure 8. The apparent activation energy (E) and the collision frequency (A) calculated from the Arrhenius plot fitting are 3.8336 kJ/mol and 1.1171 day^−1^, respectively.

### 3.6. Limitations in This Study

The strength developments were measured along the predetermined curing time and were “assumed” as reaction progress. The solid-state kinetics were initially based on empirical studies of homogenous gas-phase reactions, adopted to solid-state reactions [49]. Many theories and models have been proposed for describing specific phenomena, including geopolymerizations. It is worth mentioning that solid-state reactions have some specific features that cannot be found in homogenous reactions, such as the state of precursors (particle size and surface texture), particle interfaces, and highly varied geometric shapes. Those aspects may have a significant effect on the reaction. Thus, the generated values in this study could be applied in narrow range conditions and variables.

IR analysis has been widely proved for characterizing the aluminosilicate bonds formation, which is useful for detecting the structural changes, as a function of several variables, such as curing time and temperature, types of activators and starting materials. However, IR analysis is insufficient to describe the quantity of silicate structural units, which indicates the nature of bridging and non-bridging oxygen. Nuclear Magnetic Spectroscopy (^27^Al, ^29^Si-NMR) is needed to provide those results and the degree of connectivity of reaction products [50,51,52].

### 3.7. Circular Economy: An Approach in Coal Fly Ash and Silica Geothermal in Green Geopolymer

**Figure 9:**
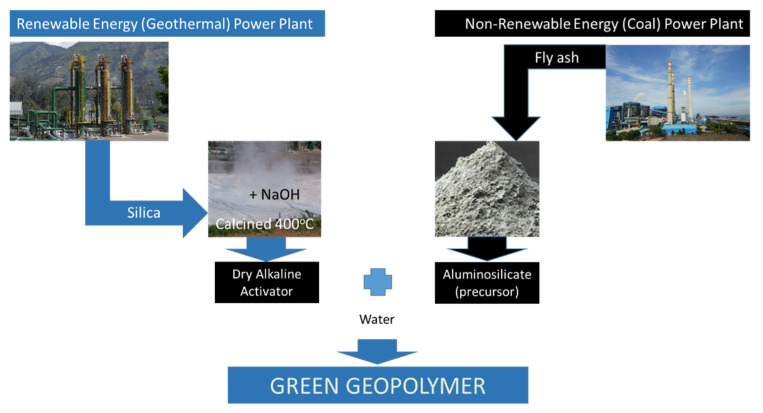
The Establishment of Circular Economy in coal and geothermal electric power generation.

Contributing approximately 7% of global anthropogenic CO_2_ emissions, the use of OPC in material construction has been minimized in recent decades [53]. The application of industrial solid waste as an alternative is prone to realization. One of the alternatives is this Green Geopolymer, which is a dry mixed geopolymer type. Aside from lower carbon dioxide emission in comparison to OPC (and Wet-Mix Geopolymer paste as reported in [23]), Green Geopolymer may also provide economic benefit. The fact that the raw materials for Green Geopolymer are from the unwanted side products of two different electric power generations will establish the circular economy conception in the energy sector, as shown in Figure 9. 

From waste to material construction products that have the potential to replace cement, green geopolymer, indeed, possesses many benefits and, most of all, the realization of circular economy to create greener technology in power plant industries.

## 4. Conclusions

Green geopolymer was successfully formed using coal fly ash from PT Paiton Energy Power Plant, Indonesia, and silica geothermal sludge from PT Geo Dipa Energi Dieng, Indonesia’s geothermal power plant, with the highest compressive strength of 22.7 MPa. The compressive strength value was obtained from the operating conditions of calcination temperature of 400 °C and curing temperature of 90 °C, with a Na_2_O/SiO_2_ ratio of 0.692. A slow heat rate is required to prevent cracking in green geopolymers during curing. The kinetics model that can describe the geopolymerization reaction with a dry activator is the diffusion kinetics model, with an average R-square value of 0.9448. The activation energy (E) is 3.8336 kJ/mol through the diffusion kinetics model, and the impact frequency (A) was 1.1171 day^−1^. The potential usage of coal fly ash and silica geothermal sludge in the green geopolymer product will accelerate the realization of circular economy conception in the energy sector of both renewable and non-renewable energies.

## Figures and Tables

**Table 1:** Comparison of wet versus dry activator in geopolymer applications.

Aspect	Wet Activator	Dry Activator	References
Temperature	Using room temperature to synthesize activator	Using room temperature to synthesize activator.	[23]
Carbon Emission	Higher emission for providing sodium silicate	No sodium silicate is needed, thus lower emission.	[28]
Reaction rate	Faster, direct ionization of pozzolan material with wet activator.	The slower needs to dissolve the solid activator first to react with pozzolan material fully.	-
Transport	Requires special and more expensive material to transport liquid activator.	Could be transported with regular OPC transporter facilities.	[23]
Setting time	Faster, only 5–58 min.	The initial setting time range from 60 to 120 min.	[29]
Safety	Contains high alkali (11.4–12.9) and high density (up to 1570 kg/m^3^ liquid) more hazardous for the worker.	It contains irritant solid, like OPC. Less hazardous for the worker.	[30]
Casting	Need to calculate the ratio of alkali activator solution and the pozzolan material.	Only need a ratio of water: cement.	[31]

**Table 2:** Geopolymerization reaction kinetics models.

Kinetics Model	Equation	References
Avrami	−ln1−α1n=kt	[33,34]
Geometric contraction	1−1−α13=kt	[34]
Diffusion	1−1−α132=k′t	[34]
Third-order	$\left(\frac{1}{2}\right)\left[{\left(1-\alpha \right)}^{-2}-1\right]=kt$	[34]

**Table 3:** Chemical composition of fly ash.

Chemical Composition	Fe	Ca	Si	Al	K	Ti	S	Others
Concentration (%wt)	40.67	29.02	18.32	6.12	2.02	1.40	1.06	1.40

**Table 4:** Oxide composition of geothermal sludge.

Oxide Composition	SiO_2_	Fe_2_O_3_	PbO	Sb_2_O_3_	CuO	Others
Concentration (%wt)	99.52	0.35	0.02	0.02	0.02	0.06

**Table 5:** Phase analysis of dry activator synthesized with NaOH and geothermal silica with a ratio of 1:1 (wt./wt.) and calcined at 400 °C.

Phase	Amorphous Phase	Sodium Metasilicate (Na_2_SiO_3_)
Concentration (%wt.)	78.86	21.14

**Table 6:** The R-square and reaction constant values are generated by fitting the degree of reaction with the kinetic reaction models.

Kinetics Model	R-Square			Reaction Constant (*k*) (Day^−1^)		
	30 °C	60 °C	90 °C	30 °C	60 °C	90 °C
Avrami	0.9565	0.9565	0.8895	0.0288	0.0392	0.0467
Geometric Contraction	0.9557	0.9557	0.8872	0.0096	0.0130	0.0155
Diffusion	0.9557	0.9557	0.8872	0.0001	0.0002	0.0002
Third order	0.9024	0.9714	0.9605	0.2423	0.2839	0.3112

## Data Availability

Not applicable.
